# Associations of Adherence to the DASH Diet and the Mediterranean Diet With All-Cause Mortality in Subjects With Various Glucose Regulation States

**DOI:** 10.3389/fnut.2022.828792

**Published:** 2022-01-27

**Authors:** Jun-Sing Wang, Wei-Ju Liu, Chia-Lin Lee

**Affiliations:** ^1^Division of Endocrinology and Metabolism, Taichung Veterans General Hospital, Taichung, Taiwan; ^2^Department of Internal Medicine, Taichung Veterans General Hospital, Taichung, Taiwan; ^3^Department of Medicine, School of Medicine, National Yang Ming Chiao Tung University, Taipei, Taiwan; ^4^College of Medicine, National Chung Hsing University, Taichung, Taiwan; ^5^Ph.D. Program in Translational Medicine, National Chung Hsing University, Taichung, Taiwan; ^6^Department of Medical Research, Taichung Veterans General Hospital, Taichung, Taiwan

**Keywords:** DASH, diabetes, Mediterranean diet, mortality, NHANES

## Abstract

**Background and Aims:**

A dietary pattern concordant with either the Dietary Approaches to Stop Hypertension (DASH) diet or the Mediterranean diet has been associated with a lower risk of all-cause mortality in general population. We investigated the associations of adherence to the DASH diet and the Mediterranean diet with all-cause mortality across three glucose regulation states (normal glucose tolerance, prediabetes, and diabetes) using data from the National Health and Nutrition Examination Survey (NHANES).

**Methods:**

Data from the NHANES participants from 1999 to 2010, including their vital status linked to the National Death Index through the end of 2011, were analyzed. Adherence to the DASH diet and the Mediterranean diet was assessed using the DASH score and the alternative Mediterranean Diet Index (aMED), respectively. Weighted Cox proportional hazards regression models were used to compare the hazard ratios for the associations of adherence (diet score >median vs. ≤ median) to the DASH diet and the Mediterranean diet with all-cause mortality.

**Results:**

A total of 28,905 participants were analyzed, and 2,598 of them had died after a median follow-up of 6.3 years. The median DASH score and aMED were 2 and 3, respectively. Adherence to the Mediterranean diet (aMED >3 vs. ≤ 3), but not the DASH diet, was associated with a lower risk of all-cause mortality (adjusted HR 0.74, 95% CI 0.66–0.83, *p* < 0.001) in the overall population. The findings were consistent across the three glucose regulation states. A joint effect of aMED >3 and DASH score >2 (adjusted HR 0.71, 95% CI 0.52–0.99, *p* = 0.042) was noted in participants with diabetes.

**Conclusions:**

Adherence to the Mediterranean diet (aMED >median) was associated with reduced all-cause mortality in a general population. For people with diabetes, a dietary pattern concordant with both the DASH diet and the Mediterranean diet (DASH score >median and aMED >median) was associated with a lower risk of mortality.

## Introduction

High diet quality is a healthy lifestyle factor that has been associated with prolonged life expectancy (1, 2). Several dietary patterns have been associated with a lower risk of all-cause mortality (3–5). Initially developed in the 1990s, the Dietary Approaches to Stop Hypertension (DASH) diet is characterized by reduced amounts of saturated fat, total fat, and cholesterol, along with high amounts of fiber, protein, and some electrolytes (potassium, magnesium, and calcium) from fruits and vegetables (6, 7). Adopting the DASH diet substantially reduced blood pressure (6, 7), with an additional effect when combined with reduced sodium intake (7, 8). The DASH score (9) was generated to assess adherence to the DASH diet, which has been associated with reduced all-cause mortality rate (3–5, 10–12).

In addition to the DASH diet, the Mediterranean diet has been recommended as a healthy dietary pattern that helps reduce blood pressure and cardiovascular (CV) risk (13). It is characterized by a relatively high intake of fruits, vegetables, nuts, legumes, whole grains, and sea food, with moderate alcohol consumption and a low intake of red/processed meat and saturated fat (14). Adopting the Mediterranean diet was effective for weight reduction, as well as lipids and glycemic control, for people with obesity (15). The alternative Mediterranean Diet Index (aMED) (16, 17) was developed to assess adherence to the Mediterranean diet, which has been associated with a lower risk of all-cause mortality in the general population (3–5, 18, 19).

Although a dietary pattern concordant with either the DASH diet or the Mediterranean diet has been associated with a lower risk of all-cause mortality (3–5), some researchers reported inconsistent findings (20–22). Moreover, the effects of adopting the DASH diet or the Mediterranean diet on all-cause mortality risk reduction in people with abnormal glucose regulation (diabetes or prediabetes) have not yet been confirmed. In this study, we investigated the associations of adherence to the DASH diet and the Mediterranean diet with all-cause mortality across the three glucose regulation states (normal glucose tolerance, prediabetes, and diabetes) using data from the National Health and Nutrition Examination Survey (NHANES).

## Materials and Methods

### Study Population

**Figure 1:**
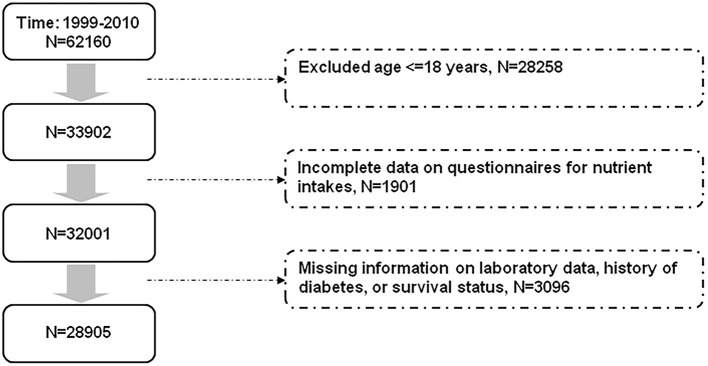
Flow diagram displaying the selection of study participants for analyses.

The NHANES consists of a series of cross-sectional examinations conducted by the National Center for Health Statistics. Participants were assessed for their health and nutritional status through anthropometric data collection, laboratory tests, and questionnaires. All participants in the NHANES provided informed consent. The dietary patterns of the participants were assessed using information from the dietary interview questionnaires (24 h dietary recalls). A second day of 24-h dietary recalls through telephone calls was conducted since 2003. For consistency, we used only the information from the single 24-h dietary recalls for analyses. This study was conducted in accordance with the Declaration of Helsinki. Our study protocol was approved by the Institutional Review Board of Taichung Veterans General Hospital, Taichung, Taiwan (approval number: CE18312A). Figure 1 shows the selection of study participants for analyses. We analyzed data of the participants in the NHANES from 1999 to 2010. After excluding participants aged ≤ 18 years and those with missing information related to nutrient intakes, laboratory data, history of diabetes, and survival status, a total of 28,905 participants were analyzed (Figure 1).

### Information on Variables for Analyses

Participants were classified as having diabetes if they reported (1) having a history of diabetes, or (2) receiving treatment with oral glucose lowering drugs or insulin, or had a fasting plasma glucose ≥ 126 mg/dl or a glycated hemoglobin (HbA1c) ≥ 6.5% (23). Among participants with no diabetes, those who had a fasting plasma glucose 100–125 mg/dl or an HbA1c 5.7–6.4% were classified as having prediabetes (23). Participants who did not fulfill the aforementioned criteria were classified as having normal glucose tolerance. Kidney function was assessed using estimated glomerular filtration rate (eGFR) determined with the Chronic Kidney Disease Epidemiology Collaboration equation (24). Chronic kidney disease was defied as having an eGFR <60 mL/min/1.73 m^2^. Daily calories consumption and the proportion of energy from carbohydrate, fat, and protein were obtained from the NHANES database. Age, sex, race/ethnicity, body mass index, history of hypertension, smoking, lipids profile, kidney function, and daily calories were compared across the three glucose regulation states, and were adjusted in the analytic models. The vital status of the NHANES participants was confirmed by linking to the National Death Index up to the end of 2011.

### Assessment of Dietary Scores

Nutrient intakes of the NHANES participants were assessed using information from the dietary interview questionnaires (24-h dietary recall). A detailed description is available online (https://wwwn.cdc.gov/nchs/nhanes/Search/DataPage.aspx?Component=Dietary&CycleBeginYear=2005). We assessed our study population's adherence to the DASH diet and the Mediterranean diet using the DASH score (9) and the aMED (16, 17), respectively, both of which had been applied to the NHANES participants (9, 17, 21, 25). The DASH score was determined based on 9 target nutrients (saturated fat, total fat, protein, cholesterol, fiber, magnesium, calcium, potassium, and sodium) (9). For each target nutrient, participants whose intake met the goal were given 1 point, while those who met the intermediate goal were given 0.5 point (maximum score = 9) (9, 10). The aMED was determined with assessments of intakes from alcohol, red and processed meat, sea food, whole grains, legumes, nuts, fruits, vegetables (except potatoes), and ratio of monounsaturated to saturated fat (16, 17). Participants whose intake was greater than the median of population intake of sea food, whole grains, legumes, nuts, fruits, vegetables (except potatoes), and ratio of monounsaturated to saturated fat were given 1 point (16, 17). For red/processed meat and alcohol, one point was assigned to those who had meat intake less than the median or moderate alcohol intake (10–25 g/day for men and 5–15 g/day for women). Participants received 0 points if the aforementioned criteria were not met (maximum score = 9). The higher the diet scores (the DASH score and the aMED), the better the concordance with the respective dietary pattern.

### Outcomes of Interest

The primary outcome in this study was all-cause mortality. All deaths after 1998 were coded following the 10th revision of the International Statistical Classification of Diseases, Injuries, and Causes of Death (ICD-10) guidelines, and were categorized into cause-specific mortality (such as CV and cancer mortality) (https://www.cdc.gov/nchs/data/nhsr/nhsr143-508.pdf). Identifying information of the NHANES participants (e.g., Social Security Number, sex, date of birth, last name, first name, …etc) were matched with the National Death Index. Participants who did not meet minimum data requirement for matching were ineligible for record linkage, and were excluded from the study population (missing information on survival status, Figure 1). All participants were divided into different groups according to the median of the DASH score and the aMED to determine whether a better adherence to the dietary pattern (>median vs. ≤ median) was associated with a lower risk of all-cause and CV mortality. We also conducted analyses in subgroups with different glucose regulation status (normal glucose tolerance, prediabetes, and diabetes).

### Statistical Analyses

All of the statistical analyses were conducted using the Statistical Analysis System survey procedures (SAS version 9.4, 2013, Cary, NC, USA). The Chi-square test and the independent sample *t*-test were used to determine the statistical significance of the between-group differences in categorical and continuous variables, respectively. All analyses were adequately weighted according to the analytic guidelines (https://wwwn.cdc.gov/nchs/nhanes/analyticguidelines.aspx). To compare the hazard ratios (HR) and 95% CI for the associations of adherence (diet score >median vs. ≤ median) to the DASH diet and the Mediterranean diet with all-cause mortality, weighted Cox proportional hazards regression models (proc surveyphreg; SAS version 9.4, 2013, Cary, NC, USA) were used with adjustment for age, sex, race, body mass index, history of hypertension, smoking, total cholesterol, chronic kidney disease, and daily energy intake. The analyses were also conducted in study participants with different glucose regulation states. In addition, joint effects of adherence to the DASH and Mediterranean diet were tested in the overall population as well as in participants with different glucose regulation states. Finally, the associations of each component of the DASH score and the aMED with all-cause mortality were examined in participants with or without diabetes. In all of the statistical analyses, a two-sided *p* value <0.05 was considered statistically significant.

## Results

The characteristics of study participants according to their glucose regulation status are shown in Table 1. Participants with diabetes were older, had a higher body mass index and systolic blood pressure, had a higher proportion of having hypertension, were less likely to smoke, had lower high-density-lipoprotein cholesterol and higher triglyceride, and had lower eGFR and daily calorie intake compared with participants with normal glucose tolerance or prediabetes. Table 2 shows the characteristics of study participants according to the DASH score (median = 2) and the aMED (median = 3) ( ≤ median vs. >median).

**Table 1:** Characteristics of study participants according to their glucose regulation status.

**Variables**	**NGT**	**Prediabetes**	**Diabetes**	***P***
Number of participants	17,082	7,408	4,415	
Age, years	41.4 (40.9–41.8)	53.5 (52.9–54.2)	58.3 (57.6–59.0)	<0.001
Male, *n* (%)	7,714 (46.2)	3,966 (53.0)	2,220 (49.1)	<0.001
Race/ethnicity, *n* (%)				<0.001
Non-Hispanic white	8,745 (73.1)	3,577 (69.1)	1,778 (64.1)	
Non-Hispanic black	2,987 (9.3)	1,548 (12.2)	1,101 (15.3)	
Mexican American/others	5,350 (17.6)	2,283 (18.7)	1,536 (20.6)	
Body mass index, kg/m^2^	27.1 (27.0–27.3)	29.9 (29.7–30.1)	32.4 (32.0–32.7)	<0.001
Hypertension, *n* (%)	3,485 (19.5)	2,986 (38.3)	2,768 (60.3)	<0.001
Smoking, *n* (%)	3,715 (52.3)	1,515 (43.6)	783 (36.0)	<0.001
Total cholesterol, mg/dl	197.7 (196.8–198.6)	206.1 (204.7–207.4)	197.5 (195.4–199.6)	<0.001
HDL cholesterol, mg/dl	54.3 (53.8–54.7)	50.7 (50.2–51.3)	47.6 (47.0–48.3)	<0.001
Triglycerides, mg/dl	132.9 (130.4–135.5)	167.8 (163.9–171.7)	210.4 (200.5–220.3)	<0.001
Fasting plasma glucose, mg/dl	86.0 (85.8–86.2)	100.1 (99.7–100.6)	149.6 (147.0–152.2)	<0.001
HbA1c, %	5.18 (5.17–5.19)	5.64 (5.63–5.66)	7.11 (7.04–7.19)	<0.001
eGFR, mL/min/1.73 m^2^	101.2 (100.5–102.0)	90.1 (89.1–91.0)	85.2 (84.1–86.3)	<0.001
Chronic kidney disease, *n* (%)^a^	738 (3.3)	810 (8.9)	808 (16.6)	<0.001
Daily calories, kcal/day	2,277 (2,255–2,298)	2,152 (2,116–2,188)	1,926 (1,883–1,969)	<0.001
% from carbohydrate	50.6 (50.4–50.9)	49.8 (49.5–50.2)	48.3 (47.8–48.7)	
% from fat	33.8 (33.6–34.0)	34.3 (33.9–34.6)	34.9 (34.5–35.4)	
% from protein	15.6 (15.5–15.7)	15.9 (15.7–16.1)	16.8 (16.6–17.1)	
DASH score	2.39 (2.35–2.42)	2.34 (2.29–2.38)	2.32 (2.26–2.38)	<0.001
aMED	3.44 (3.39–3.50)	3.41 (3.35–3.47)	3.36 (3.30–3.43)	<0.001

*Data are presented as mean (95% CI) or n (%). aMED, alternative Mediterranean Diet Index; DASH, Dietary Approaches to Stop Hypertension; eGFR, estimated glomerular filtration rate; HbA1c, glycated hemoglobin; HDL, high-density lipoprotein; NGT, normal glucose tolerance*.

a *eGFR <60 mL/min/1.73 m^2^*.

**Table 2:** Characteristics of study participants according to the DASH score and the aMED.

	**DASH score (median = 2)**			**aMED (median = 3)**		
**Variables**	**≤median**	**>median**	***P***	**≤median**	**>median**	***P***
Number of participants	14,968	13,937		15,632	13,273	
Age, years	46.1 (45.6–46.7)	46.1 (45.7–46.6)	<0.001	44.1 (43.6–44.5)	48.5 (47.9–49.1)	<0.001
Male, *n* (%)	6,932 (46.0)	6,968 (50.5)	<0.001	7,819 (50.1)	6,081 (45.8)	<0.001
Race/ethnicity, *n* (%)			<0.001			<0.001
Non-Hispanic white	7,417 (71.6)	6,683 (70.7)		7,330 (69.5)	6,770 (73.1)	
Non-Hispanic black	3,364 (12.3)	2,272 (8.8)		3,339 (11.9)	2,297 (9.2)	
Mexican American/others	4,187 (16.1)	4,982 (20.5)		4,963 (18.6)	4,206 (17.7)	
Body mass index, kg/m^2^	28.7 (28.5–28.9)	28.0 (27.8–28.1)	<0.001	28.8 (28.6–29.0)	27.9 (27.7–28.0)	<0.001
Hypertension, *n* (%)	4,889 (29.1)	4,350 (28.0)	0.063	4,837 (28.4)	4,402 (28.8)	0.514
Smoking, *n* (%)	3,275 (49.3)	2,738 (46.8)	0.035	4,106 (57.1)	1,907 (35.6)	<0.001
Total cholesterol, mg/dl	200.1 (199.2–201.0)	199.1 (198.0–200.2)	<0.001	199.3 (198.3–200.3)	200.0 (199.0–200.9)	<0.001
HDL cholesterol, mg/dl	52.6 (52.2–53.1)	52.8 (52.3–53.2)	<0.001	51.3 (50.8–51.8)	54.3 (53.9–54.8)	<0.001
Triglycerides, mg/dl	147.5 (143.9–151.2)	152.2 (148.8–155.6)	<0.001	155.8 (152.1–159.4)	142.8 (139.9–145.8)	<0.001
Fasting plasma glucose, mg/dl	96.4 (95.7–97.1)	96.3 (95.7–97.0)	<0.001	96.8 (96.1–97.4)	95.9 (95.2–96.6)	<0.001
HbA1c, %	5.51 (5.49–5.54)	5.50 (5.47–5.52)	<0.001	5.51 (5.49–5.54)	5.49 (5.47–5.52)	<0.001
eGFR, mL/min/1.73 m^2^	96.7 (95.9–97.5)	97.0 (96.3–97.7)	<0.001	98.5 (97.8–99.3)	94.9 (94.0–95.7)	<0.001
Chronic kidney disease, *n* (%)^a^	1,270 (6.4)	1,086 (5.8)	0.023	1,169 (5.6)	1,187 (6.6)	0.002
Daily calories, kcal/day	2,140 (2,120–2,160)	2,282 (2,249–2,314)	<0.001	2,169 (2,145–2,194)	2,251 (2,228–2,273)	<0.001
% from carbohydrate	46.9 (46.6–47.1)	53.8 (53.5–54.1)		49.2 (48.9–49.5)	51.3 (51.0–51.6)	
% from fat	37.9 (37.7–38.1)	29.8 (29.5–30.1)		34.8 (34.5–35.0)	33.2 (32.9–33.4)	
% from protein	15.3 (15.1–15.4)	16.4 (16.3–16.6)		16.0 (15.9–16.2)	15.6 (15.4–15.7)	

After a median follow-up of 6.3 years, 2,598 participants had died (9.9 per 1,000 person-years). The risk of all-cause mortality was higher in participants with diabetes (27.8 per 1,000 person-years) and prediabetes (15.3 per 1,000 person-years), compared with those who had normal glucose tolerance (5.9 per 1,000 person-years). Overall, adherence to the DASH diet (DASH score >2 vs. ≤ 2) was not associated with a lower risk of all-cause mortality (adjusted HR 0.93, 95% CI 0.82–1.05, *p* = 0.215, Table 3). In contrast, adherence to the Mediterranean diet (aMED >3 vs. ≤ 3) was associated with a lower risk of all-cause mortality (adjusted HR 0.74, 95% CI 0.66–0.83, *p* < 0.001, Table 3). The findings were consistent across the three glucose regulation states (*p* for interaction 0.168 and 0.777, respectively). There were no significant associations between the two dietary patterns and risk of CV mortality.

**Table 3:** Associations of adherence to DASH and Mediterranean diet with all-cause and CV mortality.

	**Adjusted HR (95% CI)^a^**	***P***	***P* for interaction**
All-cause mortality			
DASH score (>2 vs. ≤ 2)			
Overall population	0.93 (0.82–1.05)	0.215	
Normal glucose tolerance	1.02 (0.83–1.24)	0.874	0.168
Prediabetes	0.86 (0.71–1.03)	0.103	
Diabetes	0.86 (0.71–1.05)	0.147	
aMED (>3 vs. ≤ 3)			
Overall population	0.74 (0.66–0.83)	<0.001	
Normal glucose tolerance	0.75 (0.63–0.89)	0.002	0.777
Prediabetes	0.71 (0.59–0.86)	<0.001	
Diabetes	0.82 (0.66–1.03)	0.092	
CV mortality			
DASH score (>2 vs. ≤ 2)			
Overall population	1.00 (0.78–1.28)	0.995	
Normal glucose tolerance	1.17 (0.77–1.78)	0.452	0.350
Prediabetes	0.91 (0.58–1.44)	0.694	
Diabetes	0.88 (0.57–1.37)	0.569	
aMED (>3 vs. ≤ 3)			
Overall population	0.81 (0.61–1.07)	0.135	
Normal glucose tolerance	0.73 (0.50–1.09)	0.121	0.329
Prediabetes	0.76 (0.52–1.11)	0.152	
Diabetes	1.06 (0.67–1.67)	0.807	

*aMED, alternative Mediterranean Diet Index; CV, cardiovascular; DASH, Dietary Approaches to Stop Hypertension*.

a *Adjusted for age, sex, race, body mass index, history of hypertension, smoking, total cholesterol, chronic kidney disease, and daily energy intake*.

Table 4 shows the joint effects of adherence to the DASH diet and the Mediterranean diet on all-cause mortality. Compared with the reference group (DASH score ≤ 2 and aMED ≤ 3), aMED >3 (with DASH score ≤ 2 or >2) was associated with a lower risk of all-cause mortality in the overall population (both *p* < 0.001, Table 4). The findings were similar in participants with normal glucose regulation or prediabetes. Among participants with diabetes, the lower risk of all-cause mortality in those who had aMED >3 and DASH score >2 (adjusted HR 0.71, 95% CI 0.52–0.99, *p* = 0.042) was not observed in participants who had aMED >3 but DASH score ≤ 2 (adjusted HR 1.00, 95% CI 0.76–1.32, *p* = 0.995, Table 4).

**Table 4:** Joint effects of adherence to DASH and Mediterranean diet on all-cause mortality.

	**Adjusted HR (95% CI)^a^**	***P***	***P* for trend**
Overall population			
DASH score ≤ 2 and aMED ≤ 3	1 (ref)		<0.001
DASH score >2 and aMED ≤ 3	0.99 (0.84–1.18)	0.931	
DASH score ≤ 2 and aMED >3	0.76 (0.65–0.89)	<0.001	
DASH score >2 and aMED >3	0.72 (0.61–0.85)	<0.001	
Normal glucose tolerance			
DASH score ≤ 2 and aMED ≤ 3	1 (ref)		0.017
DASH score >2 and aMED ≤ 3	1.10 (0.84–1.44)	0.500	
DASH score ≤ 2 and aMED >3	0.76 (0.60–0.96)	0.024	
DASH score >2 and aMED >3	0.79 (0.61–1.02)	0.073	
Prediabetes			
DASH score ≤ 2 and aMED ≤ 3	1 (ref)		<0.001
DASH score >2 and aMED ≤ 3	0.79 (0.61–1.02)	0.065	
DASH score ≤ 2 and aMED >3	0.65 (0.51–0.82)	<0.001	
DASH score >2 and aMED >3	0.66 (0.51–0.85)	0.002	
Diabetes			
DASH score ≤ 2 and aMED ≤ 3	1 (ref)		0.047
DASH score >2 and aMED ≤ 3	1.07 (0.82–1.38)	0.626	
DASH score ≤ 2 and aMED >3	1.00 (0.76–1.32)	0.995	
DASH score >2 and aMED >3	0.71 (0.52–0.99)	0.042	

*aMED, alternative Mediterranean Diet Index; DASH, Dietary Approaches to Stop Hypertension*.

a *Adjusted for age, sex, race, body mass index, history of hypertension, smoking, total cholesterol, chronic kidney disease, and daily energy intake*.

The associations of individual components of the DASH score and the aMED with all-cause mortality in participants with or without diabetes are shown in Tables 5, 6, respectively. In general, diabetes was associated with a higher risk of mortality compared with no diabetes (score = 0 with diabetes vs. score = 0 with no diabetes [the reference group]). Examining each component of the DASH score (Table 5), adequate fiber (adjusted HR 0.70, 95% CI 0.56–0.88, *p* = 0.003), magnesium (adjusted HR 0.73, 95% CI 0.58–0.93, *p* = 0.001), or potassium (adjusted HR 0.83, 95% CI 0.67–1.03, *p* = 0.087) intake was associated with a lower risk of mortality in participants with no diabetes (score = 1 with no diabetes vs. the reference group). The risk of mortality in participants with diabetes who had adequate intake of fiber (adjusted HR 0.94, 95% CI 0.77–1.14, *p* = 0.513), magnesium (adjusted HR 1.02, 95% CI 0.78–1.32, *p* = 0.909), or potassium (adjusted HR 1.12, 95% CI 0.92–1.36, *p* = 0.269) was similar to the reference group (Table 5). Examining each component of the aMED (Table 6), similar findings were noted in participants who had a higher intake of whole grains, nuts, fruits, or vegetables.

**Table 5:** Associations of individual components of DASH score with all-cause mortality.

	**Adjusted HR (95% CI)^a^**	***P***
Saturated fat score = 0, no diabetes	1 (ref)	
Saturated fat score = 1, no diabetes	1.03 (0.86–1.23)	0.755
Saturated fat score = 0, diabetes	1.42 (1.23–1.65)	<0.001
Saturated fat score = 1, diabetes	1.20 (0.98–1.47)	0.073
Total fat score = 0, no diabetes	1 (ref)	
Total fat score = 1, no diabetes	0.99 (0.83–1.18)	0.915
Total fat score = 0, diabetes	1.39 (1.19–1.62)	<0.001
Total fat score = 1, diabetes	1.25 (1.04–1.50)	0.016
Protein score = 0, no diabetes	1 (ref)	
Protein score = 1, no diabetes	0.96 (0.79–1.15)	0.626
Protein score = 0, diabetes	1.39 (1.18–1.63)	<0.001
Protein score = 1, diabetes	1.24 (1.02–1.49)	0.030
Cholesterol score = 0, no diabetes	1 (ref)	
Cholesterol score = 1, no diabetes	1.10 (0.89–1.36)	0.387
Cholesterol score = 0, diabetes	1.46 (1.21–1.75)	<0.001
Cholesterol score = 1, diabetes	1.39 (1.16–1.66)	<0.001
Fiber score = 0, no diabetes	1 (ref)	
Fiber score = 1, no diabetes	0.70 (0.56–0.88)	0.003
Fiber score = 0, diabetes	1.35 (1.17–1.56)	<0.001
Fiber score = 1, diabetes	0.94 (0.77–1.14)	0.513
Magnesium score = 0, no diabetes	1 (ref)	
Magnesium score = 1, no diabetes	0.73 (0.58–0.93)	0.001
Magnesium score = 0, diabetes	1.35 (1.17–1.56)	<0.001
Magnesium score = 1, diabetes	1.02 (0.78–1.32)	0.909
Calcium score = 0, no diabetes	1 (ref)	
Calcium score = 1, no diabetes	0.94 (0.77–1.15)	0.535
Calcium score = 0, diabetes	1.33 (1.14–1.55)	<0.001
Calcium score = 1, diabetes	1.32 (1.09–1.60)	0.005
Potassium score = 0, no diabetes	1 (ref)	
Potassium score = 1, no diabetes	0.83 (0.67–1.03)	0.087
Potassium score = 0, diabetes	1.35 (1.16–1.57)	<0.001
Potassium score = 1, diabetes	1.12 (0.92–1.36)	0.269
Sodium score = 0, no diabetes	1 (ref)	
Sodium score = 1, no diabetes	1.44 (1.19–1.74)	<0.001
Sodium score = 0, diabetes	1.53 (1.28–1.84)	<0.001
Sodium score = 1, diabetes	1.76 (1.42–2.18)	<0.001

*DASH, Dietary Approaches to Stop Hypertension*.

a *Adjusted for age, sex, race, body mass index, history of hypertension, smoking, total cholesterol, chronic kidney disease, and daily energy intake*.

**Table 6:** Associations of individual components of aMED with all-cause mortality.

	**Adjusted HR (95% CI)^a^**	***P***
Alcohol score = 0, no diabetes	1 (ref)	
Alcohol score = 1, no diabetes	0.80 (0.58–1.10)	0.173
Alcohol score = 0, diabetes	1.35 (1.18–1.56)	<0.001
Alcohol score = 1, diabetes	0.98 (0.72–1.34)	0.909
Red/processed meat score = 0, no diabetes	1 (ref)	
Red/processed meat score = 1, no diabetes	1.00 (0.80–1.25)	0.995
Red/processed meat score = 0, diabetes	1.31 (1.07–1.60)	0.010
Red/processed meat score = 1, diabetes	1.40 (1.11–1.76)	0.005
Sea food score = 0, no diabetes	1 (ref)	
Sea food score = 1, no diabetes	0.89 (0.72–1.10)	0.283
Sea food score = 0, diabetes	1.37 (1.19–1.57)	<0.001
Sea food score = 1, diabetes	1.40 (1.11–1.76)	0.238
Whole grains score = 0, no diabetes	1 (ref)	
Whole grains score = 1, no diabetes	0.87 (0.70–1.07)	0.183
Whole grains score = 0, diabetes	1.42 (1.17–1.73)	<0.001
Whole grains score = 1, diabetes	1.12 (0.92–1.36)	0.254
Legumes score = 0, no diabetes	1 (ref)	
Legumes score = 1, no diabetes	1.10 (0.94–1.30)	0.230
Legumes score = 0, diabetes	1.40 (1.19–1.64)	<0.001
Legumes score = 1, diabetes	1.42 (1.20–1.68)	<0.001
Nuts score = 0, no diabetes	1 (ref)	
Nuts score = 1, no diabetes	0.67 (0.57–0.80)	<0.001
Nuts score = 0, diabetes	1.33 (1.15–1.54)	<0.001
Nuts score = 1, diabetes	0.94 (0.79–1.13)	0.520
Fruits score = 0, no diabetes	1 (ref)	
Fruits score = 1, no diabetes	0.80 (0.68–0.95)	0.012
Fruits score = 0, diabetes	1.24 (1.03–1.49)	0.027
Fruits score = 1, diabetes	1.14 (0.96–1.36)	0.137
Vegetables score = 0, no diabetes	1 (ref)	
Vegetables score = 1, no diabetes	0.83 (0.71–0.99)	0.033
Vegetables score = 0, diabetes	1.41 (1.19–1.66)	<0.001
Vegetables score = 1, diabetes	1.06 (0.91–1.23)	0.481
MUFA/SFA score = 0, no diabetes	1 (ref)	
MUFA/SFA score = 1, no diabetes	1.12 (0.95–1.32)	0.179
MUFA/SFA score = 0, diabetes	1.48 (1.24–1.77)	<0.001
MUFA/SFA score = 1, diabetes	1.39 (1.20–1.61)	<0.001

*aMED, alternative Mediterranean Diet Index; MUFA, monounsaturated fat; SFA, saturated fat*;

a *Adjusted for age, sex, race, body mass index, history of hypertension, smoking, total cholesterol, chronic kidney disease, and daily energy intake*.

## Discussion

In this study, we investigated the associations of adherence to the DASH diet and the Mediterranean diet with all-cause mortality in people with different glucose regulation states. We demonstrated that better adherence to the Mediterranean diet (aMED >3 vs. ≤ 3), but not the DASH diet (DASH score >2 vs. ≤ 2), was associated with a lower risk of all-cause mortality in the NHANES participants (Table 3). A joint effect of adherence to the DASH diet and the Mediterranean diet was noted in people with diabetes (Table 4). Our findings suggest that adherence to the Mediterranean diet (aMED > median) was associated with a lower risk of mortality in a general population. For people with diabetes, a lower risk of mortality was noted in those who had a dietary pattern concordant with both the DASH diet and the Mediterranean diet (DASH score >median and aMED >median).

Adherence to the DASH diet (10–12, 26) and the Mediterranean diet (18, 19, 27, 28) has been associated with reduced risk of mortality, although the results are inconsistent. For example, adherence to the DASH diet in adults with hypertension (10) has been associated with a lower risk of all-cause mortality. However, there was no significant association between the DASH scores and risk of all-cause mortality in a general population (21). The DASH diet, initially proposed in the 1990s to help treat hypertension (6, 7), was effective for blood pressure reduction (6–8). A recent meta-analysis (26) reported that adherence to the DASH diet was associated with a lower risk of all-cause mortality. However, some researchers suggested that strict adherence to the DASH diet might be necessary to attain a survival benefit (29). Unfortunately, concordance with the DASH diet was not good enough in the general population (median DASH score = 3) (21), even in patients with hypertension (mean DASH score = 2.9) (9). Overall low concordance with the DASH diet (median DASH score = 2) in the study population might help explain the null effect on all-cause mortality in our results. We stratified our study participants into three groups by their DASH scores (<2 [the reference group], 2–5, ≥ 5), and we observed that the adjusted HR [95% CI] for all-cause mortality were 1 (ref), 0.92 [0.80–1.05], and 0.80 [0.62–1.03], respectively (data not shown in Results). We speculate that maintenance of high concordance with the DASH diet is important in order to obtain a beneficial effect on outcomes (29).

In contrast, adherence to the Mediterranean diet (aMED >3 vs. ≤ 3) was associated with a lower risk of all-cause mortality in the NHANES participants. This finding was consistent across the three glucose regulation states, although there was only a modest decrease in participants with diabetes (*p* = 0.092, *p* for interaction = 0.777, Table 3). It is interesting to note that the effect of aMED >3 (vs. ≤ 3) on all-cause mortality in people with normal glucose tolerance or prediabetes was significant irrespective of the DASH score (>2 or ≤ 2) (Table 4). A joint effect was noted in people with diabetes, i.e., a lower risk of mortality was noted in those who had an aMED >3 and a DASH score >2. Greater adherence to the Mediterranean diet has been associated with improved survival (18, 19). Nevertheless, it is not yet clear whether the survival benefit is consistent across different glucose regulation states. Adherence to the Mediterranean diet has been associated with a decrease in all-cause mortality risk in people with a metabolically healthy obese phenotype, but not in those with a metabolically unhealthy obese phenotype (30). In contrast, adherence to the DASH diet was associated with a reduction in the risk of all-cause mortality in a metabolically unhealthy phenotype, but not in a metabolically healthy phenotype (31). These findings may help explain our results that a beneficial effect of aMED >3 in our participants with diabetes was noted in those who also had a DASH score >2 (Table 4).

We examined the associations of the individual components of the diet scores with all-cause mortality (Tables 5, 6). Among the components of the DASH score, an increase in fiber (32, 33), magnesium (34, 35), or potassium (36, 37) intake was associated with a reduction in the risk of all-cause mortality. It is interesting to note that a low-sodium intake (sodium score = 1, <2,400 mg/2,100 kcal diet) was associated with a higher risk of mortality in participants with or without diabetes (Table 5). A higher risk of all-cause mortality associated with low sodium excretion (approximately <4.0 g/day) has been reported (36, 37). The mean sodium intake in the NHANES participants was around 3,500 mg/2,000 kcal (38). As sodium is an essential nutrient required for normal physiology, whether a low sodium intake (<2,400 mg/2,100 kcal diet) is appropriate for CV health warrants further investigation (39). Regarding the components of aMED (Table 6), an increase in intake of whole grains (40–42), nuts (43–45), fruits (46–48), or vegetables (48, 49) was associated with a lower risk of all-cause mortality.

There were some limitations in this study. First, our analyses were based on information collected through a 24-h dietary recall interview with questionnaires. All dietary interviewers in the NHANES were required to complete a training course and to conduct supervised practice interviews before working independently to minimize data collection bias. Moreover, retraining sessions were conducted annually to reinforce the proper protocols. Thus, the quality of dietary information was satisfactory for research use. Nevertheless, long-term adherence to the dietary patterns was not addressed in this study. This issue should be taken into account when interpreting our results. Second, this was a cohort study and the between-group differences in baseline characteristics might have confounded our results. Ideally, the effect of a healthy dietary pattern on risk of mortality in patients with diabetes or prediabetes should be investigated in a randomized trial with long-term follow-up. Our findings provide novel insights into the nutritional recommendations for people with abnormal glucose regulation.

In conclusion, adherence to the Mediterranean diet (aMED > median) was associated with a lower risk of all-cause mortality in a general population. For people with diabetes, the benefit was noted in those whose dietary pattern was concordant with both the DASH diet and the Mediterranean diet (DASH score >median and aMED >median). Higher intake of fiber, magnesium, potassium, whole grains, nuts, fruits, and vegetables was associated with a lower risk of mortality in people with or without diabetes.

## Data Availability Statement

The datasets presented in this study can be found in online repositories. The names of the repository/repositories and accession number(s) can be found below: https://www.cdc.gov/nchs/nhanes/index.htm.

## Ethics Statement

The studies involving human participants were reviewed and approved by the Institutional Review Board of Taichung Veterans General Hospital, Taichung, Taiwan (Approval Number: CE18312A). The patients/participants provided their written informed consent to participate in this study.

## Author Contributions

C-LL and J-SW contributed to the conception, design of the study, interpretation of data, and wrote the first draft of the manuscript. C-LL and W-JL contributed to the acquisition and analysis of data. WJ-L revised the manuscript critically for important intellectual content. All of the authors reviewed and approved the final version of the manuscript to be published.

## Funding

This work was supported by Taichung Veterans General Hospital, Taichung, Taiwan [Grant Numbers TCVGH-1083505C, 2019; TCVGH-1093504C, 2020; TCVGH-1103502C, TCVGH-1103504C, and TCVGH-1107305D, 2021]. The sponsor of this study was not involved in the study design; in the collection, analysis and interpretation of data; in the writing of the report; or in the decision to submit the article for publication.

## Conflict of Interest

The authors declare that the research was conducted in the absence of any commercial or financial relationships that could be construed as a potential conflict of interest.

## Publisher's Note

All claims expressed in this article are solely those of the authors and do not necessarily represent those of their affiliated organizations, or those of the publisher, the editors and the reviewers. Any product that may be evaluated in this article, or claim that may be made by its manufacturer, is not guaranteed or endorsed by the publisher.

## References

[1] Li Y Pan A Wang DD Liu X Dhana K Franco OH. Impact of healthy lifestyle factors on life expectancies in the US Population. Circulation. (2018) 138:345–55. 10.1161/CIRCULATIONAHA.117.032047 29712712 PMC6207481

[2] Li Y Schoufour J Wang DD Dhana K Pan A Liu X. Healthy lifestyle and life expectancy free of cancer, cardiovascular disease, and type 2 diabetes: prospective cohort study. BMJ. (2020) 368:l6669. 10.1136/bmj.l6669 31915124 PMC7190036

[3] Neelakantan N Koh WP Yuan JM van Dam RM. Diet-quality indexes are associated with a lower risk of cardiovascular, respiratory, and all-cause mortality among Chinese adults. J Nutr. (2018) 148:1323–32. 10.1093/jn/nxy094 29982724 PMC6075575

[4] Hu EA Steffen LM Coresh J Appel LJ Rebholz CM. Adherence to the healthy eating index-2015 and other dietary patterns may reduce risk of cardiovascular disease, cardiovascular mortality, and all-cause mortality. J Nutr. (2020) 150:312–21. 10.1093/jn/nxz218 31529069 PMC7373820

[5] Sotos-Prieto M Bhupathiraju SN Mattei J Fung TT Li Y Pan A. Association of changes in diet quality with total and cause-specific mortality. n Engl J Med. (2017) 377:143–53. 10.1056/NEJMoa1613502 28700845 PMC5589446

[6] Appel LJ Moore TJ Obarzanek E Vollmer WM Svetkey LP Sacks FM. A clinical trial of the effects of dietary patterns on blood pressure. DASH Collaborative Research Group n Engl J Med. (1997) 336:1117–24. 10.1056/NEJM199704173361601 9099655

[7] Sacks FM Svetkey LP Vollmer WM Appel LJ Bray GA Harsha D. DASH-Sodium Collaborative Research Group. Effects on blood pressure of reduced dietary sodium and the Dietary Approaches to Stop Hypertension (DASH) diet. (2001) n Engl J Med. 344:3–10. 10.1056/NEJM200101043440101 11136953

[8] Vollmer WM Sacks FM Ard J Appel LJ Bray GA Simons-Morton DG. DASH-Sodium Trial Collaborative Research Group. Effects of diet and sodium intake on blood pressure:subgroup analysis of the DASH-sodium trial. Ann Intern Med. (2001) 135:1019-1028. 10.7326/0003-4819-135-12-200112180-00005 11747380

[9] Mellen PB Gao SK Vitolins MZ Goff DC Jr. Deteriorating dietary habits among adults with hypertension: DASH dietary accordance, NHANES 1988-1994 and 1999-2004. Arch Intern Med. (2008) 168:308–14. 10.1001/archinternmed.2007.119 18268173

[10] Parikh A Lipsitz SR Natarajan S. Association between a DASH-like diet and mortality in adults with hypertension: findings from a population-based follow-up study. Am J Hypertens. (2009) 22:409–16. 10.1038/ajh.2009.10 19197247

[11] Yu D Zhang X Xiang YB Yang G Li H Gao YT. Adherence to dietary guidelines and mortality: a report from prospective cohort studies of 134,000 Chinese adults in urban Shanghai. Am J Clin Nutr. (2014) 100:693–700. 10.3945/ajcn.113.079194 24944055 PMC4095665

[12] Mokhtari Z Sharafkhah M Poustchi H Sepanlou SG Khoshnia M Gharavi A. Adherence to the dietary approaches to stop hypertension (DASH) diet and risk of total and cause-specific mortality:results from the golestan cohort study. Int J Epidemiol. (2019) 48:1824–38. 10.1093/ije/dyz079 31056682 PMC6929526

[13] Barone Gibbs B Hivert MF Jerome GJ Kraus WE Rosenkranz SK Schorr EN. American heart association council on lifestyle and cardiometabolic health; council on cardiovascular and stroke nursing; and council on clinical cardiology. physical activity as a critical component of first-line treatment for elevated blood pressure or cholesterol: who, what, and how?:a scientific statement from the American Heart Association. Hypertension. (2021) 78:e26-e37. 10.1161/HYP.0000000000000196 34074137

[14] Davis C Bryan J Hodgson J Murphy K. Definition of the Mediterranean Diet:a Literature Review. Nutrients. (2015) 7:9139–53. 10.3390/nu7115459 26556369 PMC4663587

[15] Shai I Schwarzfuchs D Henkin Y Shahar DR Witkow S Greenberg I. Dietary Intervention Randomized Controlled Trial (DIRECT) group. weight loss with a low-carbohydrate, mediterranean, or low-fat diet. n Engl J Med. (2008) 359:229–41. 10.1056/NEJMoa0708681 18635428

[16] Fung TT McCullough ML Newby PK Manson JE Meigs JB Rifai N. Diet-quality scores and plasma concentrations of markers of inflammation and endothelial dysfunction. Am J Clin Nutr. (2005) 82:163–73. 10.1093/ajcn/82.1.163 16002815

[17] Ha K Kim K Sakaki JR Chun OK. Relative Validity of Dietary Total Antioxidant Capacity for Predicting All-Cause Mortality in Comparison to Diet Quality Indexes in US Adults. Nutrients. (2020) 12:1210. 10.3390/nu12051210 32344879 PMC7282024

[18] Trichopoulou A Costacou T Bamia C Trichopoulos D. Adherence to a Mediterranean diet and survival in a Greek population. n Engl J Med. (2003) 348:2599–608. 10.1056/NEJMoa025039 12826634

[19] Trichopoulou A Bamia C Trichopoulos D. Anatomy of health effects of Mediterranean diet:Greek EPIC prospective cohort study. BMJ. (2009) 338:b2337. 10.1136/bmj.b2337 19549997 PMC3272659

[20] Shah NS Leonard D Finley CE Rodriguez F Sarraju A Barlow CE. Dietary Patterns and Long-Term Survival:A Retrospective Study of Healthy Primary Care Patients. Am J Med. (2018) 131:48–55. 10.1016/j.amjmed.2017.08.010 28860032

[21] Beydoun HA Huang S Beydoun MA Hossain S Zonderman AB. Mediating-moderating effect of allostatic load on the association between dietary approaches to stop hypertension diet and all-cause and cause-specific mortality:2001-2010 national health and nutrition examination surveys. Nutrients. (2019) 11:2311. 10.3390/nu11102311 31569527 PMC6836046

[22] Zhong VW Ning H Van Horn L Carnethon MR Wilkins JT Lloyd-Jones DM. Diet quality and long-term absolute risks for incident cardiovascular disease and mortality. Am J Med. (2021) 134:490–8. 10.1016/j.amjmed.2020.08.012 32941845 PMC7956066

[23] American Diabetes Association. Classification and diagnosis of diabetes:standards of medical care in diabetes-2020. Diabetes Care. (2020). 43:S14–31. 10.2337/dc20-S002 31862745

[24] Levey AS Stevens LA Schmid CH Zhang YL Castro AF 3rd Feldman HI. A new equation to estimate glomerular filtration rate. Ann Intern Med. (2009) 150:604–12. 10.7326/0003-4819-150-9-200905050-00006 19414839 PMC2763564

[25] Lee CL Liu WJ Wang JS. Associations of low-carbohydrate and low-fat intakes with all-cause mortality in subjects with prediabetes with and without insulin resistance. Clin Nutr. (2021) 40:3601–7. 10.1016/j.clnu.2020.12.019 33390277

[26] Soltani S Arablou T Jayedi A Salehi-Abargouei A. Adherence to the dietary approaches to stop hypertension (DASH) diet in relation to all-cause and cause-specific mortality: a systematic review and dose-response meta-analysis of prospective cohort studies. Nutr J. (2020) 19:37. 10.1186/s12937-020-00554-8 32321528 PMC7178992

[27] Lopez-Garcia E Rodriguez-Artalejo F Li TY Fung TT Li S Willett WC Rimm EB. The Mediterranean-style dietary pattern and mortality among men and women with cardiovascular disease. Am J Clin Nutr. (2014) 99:172–80. 10.3945/ajcn.113.068106 24172306 PMC3862454

[28] Sofi F Abbate R Gensini GF Casini A. Accruing evidence on benefits of adherence to the Mediterranean diet on health: an updated systematic review and meta-analysis. Am J Clin Nutr. (2010) 92:1189–96. 10.3945/ajcn.2010.29673 20810976

[29] Folsom AR Parker ED Harnack LJ. Degree of concordance with DASH diet guidelines and incidence of hypertension and fatal cardiovascular disease. Am J Hypertens. (2007) 20:225–32. 10.1016/j.amjhyper.2006.09.003 17324731 PMC2020811

[30] Park YM Steck SE Fung TT Zhang J Hazlett LJ Han K. Mediterranean diet and mortality risk in metabolically healthy obese and metabolically unhealthy obese phenotypes. Int J Obes. (2016) 40:1541–9. 10.1038/ijo.2016.114 27339604

[31] Park YM Fung TT Steck SE Zhang J Hazlett LJ Han K. Diet quality and mortality risk in metabolically obese normal-weight adults. Mayo Clin Proc. (2016) 91:1372–83. 10.1016/j.mayocp.2016.06.022 27712636

[32] Streppel MT Ocké MC Boshuizen HC Kok FJ Kromhout D. Dietary fiber intake in relation to coronary heart disease and all-cause mortality over 40 y:the Zutphen Study. Am J Clin Nutr. (2008) 88:1119–25. 10.1093/ajcn/88.4.1119 18842802

[33] Park Y Subar AF Hollenbeck A Schatzkin A. Dietary fiber intake and mortality in the NIH-AARP diet and health study. Arch Intern Med. (2011) 171:1061–8. 10.1001/archinternmed.2011.18 21321288 PMC3513325

[34] Guasch-Ferré M Bulló M Estruch R Corella D Martínez-González MA Ros E. PREDIMED Study Group. Dietary magnesium intake is inversely associated with mortality in adults at high cardiovascular disease risk. J Nutr. (2014) 144:55–60. 10.3945/jn.113.183012 24259558

[35] Chen F Du M Blumberg JB Ho Chui KK Ruan M Rogers G. Association among dietary supplement use, nutrient intake, mortality among U.S. Adults: a cohort study. Ann Intern Med. (2019) 170:604–13. 10.7326/M18-2478 30959527 PMC6736694

[36] O'Donnell M Mente A Rangarajan S McQueen MJ Wang X Liu L. PURE Investigators. Urinary sodium and potassium excretion, mortality, and cardiovascular events. n Engl J Med. (2014) 371:612–23. 10.1056/NEJMoa1311889 25119607

[37] O'Donnell M Mente A Rangarajan S McQueen MJ O'Leary N Yin L. PURE Investigators. Joint association of urinary sodium and potassium excretion with cardiovascular events and mortality:prospective cohort study. BMJ. (2019) 364:l772. 10.1136/bmj.l772 30867146 PMC6415648

[38] McClure ST Schlechter H Oh S White K Wu B Pilla SJ. Dietary intake of adults with and without diabetes:results from NHANES 2013-2016. BMJ Open Diabetes Res Care. (2020) 8:e001681. 10.1136/bmjdrc-2020-001681 33099509 PMC7590352

[39] O'Donnell M Mente A Alderman MH Brady AJB Diaz R Gupta R. Salt and cardiovascular disease: insufficient evidence to recommend low sodium intake. Eur Heart J. (2020) 41:3363–73. 10.1093/eurheartj/ehaa586 33011774

[40] Huang T Xu M Lee A Cho S Qi L. Consumption of whole grains and cereal fiber and total and cause-specific mortality: prospective analysis of 367,442 individuals. BMC Med. (2015) 13:59. 10.1186/s12916-015-0294-7 25858689 PMC4371798

[41] Wu H Flint AJ Qi Q van Dam RM Sampson LA Rimm EB. Association between dietary whole grain intake and risk of mortality: two large prospective studies in US men and women. JAMA Intern Med. (2015) 175:373–84. 10.1001/jamainternmed.2014.6283 25559238 PMC4429593

[42] Zhang B Zhao Q Guo W Bao W Wang X. Association of whole grain intake with all-cause, cardiovascular, and cancer mortality:a systematic review and dose-response meta-analysis from prospective cohort studies. Eur J Clin Nutr. (2018) 72:57–65. 10.1038/ejcn.2017.149 29091078

[43] Bao Y Han J Hu FB Giovannucci EL Stampfer MJ Willett WC. Association of nut consumption with total and cause-specific mortality. n Engl J Med. (2013) 369:2001–11. 10.1056/NEJMoa1307352 24256379 PMC3931001

[44] Guasch-Ferré M Bulló M Martínez-González MÁ Ros E Corella D Estruch R. PREDIMED study group. Frequency of nut consumption and mortality risk in the PREDIMED nutrition intervention trial. BMC Med. (2013) 11:164. 10.1186/1741-7015-11-164 23866098 PMC3738153

[45] Luu HN Blot WJ Xiang YB Cai H Hargreaves MK Li H. Prospective evaluation of the association of nut/peanut consumption with total and cause-specific mortality. JAMA Intern Med. (2015) 175:755–66. 10.1001/jamainternmed.2014.8347 25730101 PMC4474488

[46] Key TJ Thorogood M Appleby PN Burr ML. Dietary habits and mortality in 11,000 vegetarians and health conscious people:results of a 17 year follow up. BMJ. (1996) 313:775–9. 10.1136/bmj.313.7060.775 8842068 PMC2352199

[47] Strandhagen E Hansson PO Bosaeus I Isaksson B Eriksson H. High fruit intake may reduce mortality among middle-aged and elderly men. The Study of Men Born in 1913. Eur J Clin Nutr. (2000) 54:337–41. 10.1038/sj.ejcn.1600959 10745285

[48] Hjartåker A Knudsen MD Tretli S Weiderpass E. Consumption of berries, fruits and vegetables and mortality among 10,000 Norwegian men followed for four decades. Eur J Nutr. (2015) 54:599–608. 10.1007/s00394-014-0741-9 25087093

[49] Zhang X Shu XO Xiang YB Yang G Li H Gao J. Cruciferous vegetable consumption is associated with a reduced risk of total and cardiovascular disease mortality. Am J Clin Nutr. (2011) 94:240–6. 10.3945/ajcn.110.009340 21593509 PMC3127519

